# CERES: Cluster‑enabled regression of extract signatures for discovery of NRF2 activators in *Centella asiatica* by ion-mobility mass spectrometry, k-medoids clustering and ensemble Lasso regression

**DOI:** 10.1371/journal.pone.0354664

**Published:** 2026-08-06

**Authors:** Luke C. Marney, Jaewoo Choi, Vera Alenicheva, Kadine Cabey, Tobias Beck, Elizabeth Milner, Nora E. Gray, Amala Soumyanath, Claudia S. Maier, Jan F. Stevens, Kevin S. Brown

**Affiliations:** 1 BENFRA Botanical Dietary Supplements Research Center, Oregon Health & Science University, Portland, Oregon, United States of America; 2 Department of Chemistry, Oregon State University, Corvallis, Oregon, United States of America; 3 Department of Neurology, Oregon Health & Science University, Portland, Oregon, United States of America; 4 School of Chemical, Biological, and Environmental Engineering, Oregon State University, Corvallis, Oregon, United States of America; 5 Department of Pharmaceutical Sciences, Oregon State University, Corvallis, Oregon, United States of America; 6 Linus Pauling Institute, Oregon State University, Corvallis, Oregon, United States of America; Dr. Anjali Chatterji Regional Research Institute for Homeopathy, INDIA

## Abstract

Discovery of bioactive phytochemicals from botanical extracts is a pivotal step for the modernization of traditional medicine and in the discovery of novel pharmaceuticals. However, traditional bioassay-guided fractionation is laborious and often leads to the re-identification of known compounds. To address these challenges, we have developed a novel discovery pipeline, called CERES (Cluster-Enabled Regression of Extract Signatures) that combines preparative column chromatography-based fractionation and bioactivity assessment, followed by loop-injection ion-mobility mass spectrometry, with machine learning. We apply cluster-based feature reduction and regularized regression models for rapid identification of bioactive phytochemicals. This approach reduces the need for analytical chromatographic separation in early dereplication stages, reducing bias and increasing speed, while remaining complementary to downstream structural confirmation workflows. By grouping mass spectrometry features into clusters representing different chemical entities derived from the same molecular species, the computational approach effectively reduces dimensionality and improves performance and interpretability of model results. The computational approach was successfully applied here to identify bioactive compounds in fractions of *Centella asiatica* water extract and uncovered bioactive mass-to-charge signals linked to known chemical features as well as new low-abundant, yet to be annotated signals to be investigated in further studies. This innovative approach offers a powerful and efficient way to advance the integration of traditional medicine with evidence-based practice by leveraging the power of ion-mobility mass spectrometry and machine learning.

## Introduction

Phytochemicals derived from botanical extracts are a rich source of pharmaceutically active chemical components. However, identifying specialized metabolites in botanicals that elicit bioactivity in bioassays, preclinical *in vivo* models, or humans has historically relied on bioassay-guided fractionation and is a tedious and time-consuming process [1,2]. This approach requires the separation of phytochemicals based on physicochemical properties and assessing their bioactivity in a step-by-step methodology to sequentially isolate suspected bioactive compounds. Sequential steps of purification and assaying may result ultimately in the isolation of the bioactive compound, only to find that this compound has been already discovered [1–5], a set-back in drug discovery programs. There is an urgent need to accelerate the discovery of bioactive natural products and support early dereplication. Additionally, compounds can degrade or get lost during traditional exhaustive fractionation [6], and bioactivity may require the co-presence of other phytochemical compounds, a phenomenon sometimes known as entourage effects [7]. Thus, the identification of active compounds within active mixtures may be preferable to the goal of obtaining purified single active compounds.

New methods for bioactive metabolite discovery and characterization are particularly important if we want to integrate natural products into standard medical practice. As per the National Center for Complementary and Integrative Health (NCCIH), approximately 17.7% of U.S. adults reported using natural products in a given month (National Health Interview Survey 2012 | NCCIH (nih.gov)). Despite this prevalence, moving these practices into the realm of evidence-based medicine remains a significant challenge [8]. Natural product integration into medical practice is traditionally based on cultural and ethnobotanical traditions, which often rely on folk knowledge and practices [9–21]. Moving to evidence-based usage of natural products requires rigorous establishment of the bioactivity and toxicity profiles of specific natural products, which is currently hindered by their inherent phytochemical complexity and the laborious effort to isolate and identify specific bioactive or toxic elements for product standardization and clinical testing.

Methods capable of addressing this need for better metabolite discovery and characterization are emerging. Combining high-throughput bioassay screening with loop-injection mass spectrometry analysis of fractions enables the detection of mass spectrometry signals associated with therapeutic bioactivity (and toxicity) without the need for extensive fractionation and compound isolation. Regularized regression models can be used [22–25] and are capable of correctly identifying the bioactive constituents in complex botanical extracts from high resolution mass spectrometry profiles of extract fractions [26]. Our approach builds on these advances while aligning with a broader trend in ML-assisted metabolomics toward principled, data-driven feature reduction, replacing heuristic signal-to-noise or adduct-based filtering with strategies that control feature dimensionality while retaining chemically interpretable signals rather than aggregate representations.

However, challenges remain, particularly with respect to the “many-to-one” mapping problem in mass spectrometry data analysis. Electrospray ionization mass spectrometry results in highly complex data consisting of multiple adducts, isotopologues, and in-source fragment ions of each chemical component [27]. The result is a highly correlated dataset where multiple mass-to-charge signals represent a single molecule detected in a variety of forms [28]. Combination of adducts (sometimes referred to as declustering) and deisotoping of molecular features is often accomplished by vendor or open-source software packages, yet these methods ultimately rely on chromatographic coelution of features to establish which signals to combine or remove [29–33], or they model the isotopic abundance and/or predict commonly occurring adducts *in silico* [34]. Loop-injection mass spectrometry increases the throughput of investigations as well as reduces chemical biases associated with selecting chromatographic separation, but it has no separation dimension that would allow coelution analysis for the combination of adducts and isotopes. It would be advantageous to develop unbiased methods for chemical feature reduction in this modality. Moreover, verification of these feature reduction algorithms is ultimately performed for known chemical components of an optimal chromatographic separation. Removing the chromatographic separation dimension removes the bias a selected separation method would cause, but the problem of effectively collapsing mass spectrometry isoforms remains. In this study, we describe a novel processing pipeline that can detect signals from novel compounds along with more familiar components that act as positive controls.

We want a feature reduction method that is tunable (in terms of how much reduction is done), effective for loop-injection MS, and serves as effective input to the modeling step. We use clustering to group features by across-fraction similarity, and we show in this study that clustering based on across-fraction similarity is highly effective at combining the kinds of molecules (adducts, etc.) that we want to group. We choose k-medoids [35] as our clustering method because the prototypes (reduced features/mass peaks) that it produces are signals from actual compounds in the separation, not averages over cluster members that do not correspond to any actual compound’s signal, as in the more widely used k-means algorithm [36]. By pairing the clustering step with ensemble Lasso regressions for determination of chemical bioactivity, we successfully address the challenges associated with phytochemical complexity, limitations of loop injection mass spectrometry (where the number of mass spectrometry features is much larger than the number of fractions tested), and non-specific bioassay effects. This methodology extends and improves upon our previously successful computational discovery pipeline which used a conservative signal-to-noise filter and the Elastic Net for bioactivity to mass spectrometry signal regression analysis [26].

In k-medoids, like many clustering algorithms, the number of desired clusters is a free parameter. We present a model-driven approach to selecting this parameter, which controls the degree of mass spectrometry feature aggregation. The prediction models we use are specifically selected to be good at removing irrelevant mass spec features in the data, but their performance still degrades as the number of features (p) grows relative to the number of measured fractions (n). Thus, in previous work [26,37] we used rather aggressive signal-to-noise filtration and vendor available adduct combination algorithms (termed declustering), performed by proprietary software, to obtain a $\frac{p}{n}$ ratio of between 1 and 6. However, an arbitrary clustering scheme is suboptimal in terms of downstream prediction model quality. Standard clustering methods are not focused on a particular target p/n. A better approach would be to obtain systematic information about prediction model breakdown as a function of $\frac{p}{n}$ and then choose as many features as possible while still ensuring good model performance. In this study we perform a systematic comparison of several regularized regression models [24,38,39] to determine how they behave as $\frac{p}{n}$ increases, which: i) helps us select the best performing algorithm for the problem and ii) gives us a target number of features for reduction and hence a reasonable range for the number of clusters. We show that this is a powerful, flexible approach that succeeds in describing the bioactivity of clustered molecular features based on known masses from prior studies of *Centella asiatica.* Moreover, we can discover new bioactive mass-to-charge signals and determine their relationship to other known mass spectrometry signals, which yields information about the putative identity of those compounds [40,41].

Our analysis pipeline, Cluster‑Enabled Regression of Extract Signatures (CERES, https://github.com/kevinbrownlab/ceres) is applicable to other bio- and toxicological assays where mass spectrometry signals need to be correlated to assay measurements in a high-throughput and unbiased manner. Our methodological advancements not only streamline the process of identifying active compounds but also enhance our regression model’s ability to predict and discover bioactive compounds with greater accuracy despite the inherent variability in natural products. By addressing these challenges, this study contributes to the broader goal of integrating cultural and ethnobotanical medicine into evidence-based practice, ultimately advancing the evidence hierarchy for natural product therapeutics. Our approach also has significant implications for improving the safety and efficacy of natural products, paving the way for more reliable and scalable analytical methods and ultimately more effective discovery of novel therapeutic phytochemicals. Importantly, CERES is designed to complement and accelerate the early stages of conventional bioassay-guided workflows by rapidly prioritizing candidate bioactive fractions and compound identities for downstream structural investigation, rather than replacing established methods for compound isolation and characterization.

## Materials and methods

### Chemicals and reagents

LC-MS grade methanol, water, and formic acid were purchased from Fisher Scientific (Hampton, NH, USA). Twelve non-deuterated, authentic standards were purchased from Cayman Chemical (Ann Arbor, MI, USA). Names according to the manufacturer were 4-O-caffeoylquinic acid (cryptochlorogenic acid, 4-CQA), 5-O-caffeoylquinic acid (neochlorogenic acid, 5-CQA), 1,3-dicaffeoylquinic acid (1,3-DiCQA), 1,5-dicaffeoylquinic acid (1,5-DiCQA), 3,4-dicaffeoylquinic acid (isochlorogenic acid B, 3,4-DiCQA), 4,5-dicaffeoylquinic acid (isochlorogenic acid C, 4,5-DiCQA) and madecassoside (MS), of purity ≥ 98%; and 3-O-caffeoylquinic acid (chlorogenic acid, 3-CQA), 3,5-dicaffeoylquinic acid (isochlorogenic acid A, 3,5-DiCQA), asiaticoside (AS), madecassic acid (MA) and asiatic acid (AA), of purity ≥ 95%. Identity was verified by NMR at Oregon State University as described previously [41]. tert-Butyl hydroquinone was purchased from Sigma. NRF2/ARE Luciferase Reporter HepG2 Stable Cell Line was purchased from Signosis.

### Preparation of an aqueous extract of *Centella asiatica*

*Centella asiatica* water (CAW15) extract was prepared as previously described [40]. Briefly, dried *Centella asiatica* obtained through Oregon’s Wild Harvest (Redmond, OR, USA) was extracted by boiling dried aerial parts of the plant (stems, leaves and flowers, but not roots; 80 grams per 1 L of deionized water) for 1.5 h, replacing lost water as needed, then cooling for 30 min, and filtering to remove plant debris. The aqueous extract was freeze-dried (yield 33% w/w of original plant material) and stored at −20 °C until use. A voucher sample of this extract is stored at the BENFRA Center laboratory under code number BEN-CAW-15. A voucher sample of the *Centella asiatica* plant material is stored at the Oregon State University herbarium (OSC-V-265,416).

### Extract fractionation

Initially 25 g of dried CAW15 aqueous extract was suspended in 72 mL water and ethanol (equal parts). The sample was divided into 6 aliquots, centrifuged at 3000 rpm for 20 minutes and the supernatants collected. Supernatants were dried under nitrogen stream (removing enough ethanol for freezing) then lyophilized to a powder. A final 11.77 g of material was recovered. From the recovered, water-ethanol soluble, dried material, 6 g was dissolved in 12 mL of methanol (schematic of preparation found in S1 Fig). This methanol solution was loaded onto an LH-20 column (3 × 24 in). Column chromatography was performed with methanol as mobile phase and fractions collected using an HBI fraction collector LC 200. The fractionation resulted in 156 crude fractions. Fractions were dried under a nitrogen stream, weighed (mass distributions shown in Supplemental Fig. 3), and UV absorbance at 325 nm recorded prior to drying (Supplemental Fig. 4). Fractions were combined into 40 final fractions based on mass distribution, targeting equal final mass concentrations (375 ng/mL). Combined fractions were reconstituted in water for bioassay and mass spectrometry analysis.

### Nuclear factor erythroid 2-related factor 2 antioxidant response elements (NRF2/ARE) bioassay

The NRF2/ARE Luciferase Reporter HepG2 cell line was thawed and seeded onto white and clear 384 well plates at 12,500 cells per well using a 16-channel pipette at 50 µL/well with Eagle’s Minimum Essential Medium (EMEM) supplemented with 10% Fetal Bovine Serum and Hygromycin B (OR Penn Strep). The cells were incubated at 37℃ with 5% CO2 for 24 hours. Two 384-well plates were prepared, one plate for the NRf2/ARE assay and one plate for the Cell Titer Glo assay. Cells were exposed to the final 40 water fractions (two doses of 375 µg/mL) to a total concentration (on cells) of 750 µg/mL and in triplicate and the controls included tert-butylhydroquinone (BHQ) and pure water. Treatment samples (tBHQ, water, or extract fractions) were prepared before the experimental assay and frozen until ready for use. Samples were diluted with dH2O with Triton X-100 prior to dispensing, a nonionic surfactant that maintains cell viability. Cell dosing was completed using the HP D300e Digital Dispenser protocol in triplicate for treatments and in triplicate for controls with the T8 + aqueous dispensing cassettes. Sample layout was determined using HP D300e protocol leaving column 1, 23, and 24 empty to account for evaporation. The cells were incubated at 37℃ with 5% CO2 for 24 hours. All analyses were performed on a single extract batch (BEN-CAW-15); the bioassay was performed with three technical replicates per fraction.

### Mass spectrometry analysis of extract and extract fractions

Mass spectrometry was carried out on a Synapt G2 equipped with a Waters I-class Acquity UPLC and a flow-through needle for injection. Each of the final 40 fractions were diluted in 1:1000 in methanol containing 1 µg/mL digoxin-d3 internal standard. A VanGuard SunFire® C8 5 µm guard cartridge fitted in a VanGuard Cartridge Holder was used and a simple switching gradient with starting conditions of 98% water + 0.1% formic acid for 0.1 min followed by 98% methanol with 0.1% formic acid was used to transfer sample to the mass spectrometer. Ion mobility was operated at 90 mL/min nitrogen gas. High resolution TOFMS analysis was conducted in negative electrospray ionization mode acquiring from 100-1300 m/z and lock mass (leucine enkephalin) was continuously infused during the analysis for mass correction and verified to be < 4 ppm for each fraction following lock mass correction. Prior to mass spectrometry analysis, m/z was calibrated using NaF infusion. Mass spectrometry analysis was performed as a single acquisition per fraction.

Follow up analysis of select fractions and starting material for identity verification was performed with a Shimadzu Nexera UHPLC system connected to an AB SCIEX TripleTOF® 5600 mass spectrometer (QToF) equipped with a Turbo V ionization source operated in negative electrospray ionization (ESI) mode. The mass spectrometer was equipped with a calibrant delivery system and recalibrated every 2 hours. Chromatographic separation was achieved using an Inertsil Phenyl-3 column (2.1 × 150 mm, 100 Å, 2 μm; GL Sciences, Torrance, CA, United States). The injection volume was 3 μL. Gradient elution was performed as previously described [41]. Data-dependent acquisition was conducted using negative ionization mode. The following settings were used: full scan with ion accumulation of 250 ms, followed by a dynamic MS/MS selection of the four most intense ions with 100 ms accumulation; after three MS/MS acquisitions the precursor ions (fragmented) were excluded for 30 s; collision energy was 35 V with collision energy spread (CES) of 10 V ramped through each MS/MS scan using a range of m/z 70–1,100.

### Mass spectral conversion, calibration, merging and filtration

Mass spectra files were converted to mzML using proteowizard [42] and individual mass spectra for each fraction were produced by utilizing mzR and xcms raw data methods to collect all spectra in a 30 second time window for the ion-mobility and TOFMS acquisition [43]. An average spectrum for each fraction was calculated and each fraction’s spectrum was lock mass corrected, masses rounded to 0.001 Da, blank subtracted, merged, and signal-to-noise (S/N) filtered into one common matrix for further analysis (https://github.com/kevinbrownlab/ceres). A low S/N filter was applied to preserve low intensity signals.

### K-medoids Clustering

K-medoids [35] clustering was performed on the merged, lock-mass corrected, blank subtracted, and S/N filtered mass spectrometry signals for each fraction. K-medoids was performed in Python with the kmedoids package from scikit-learn-extra (https://scikit-learn-extra.readthedocs.io/en/stable/). As input to the subsequent modeling step, the original set of pre-clustered features was replaced with a smaller number of features, which are the cluster medoids. The advantage of k-medoids over the more widely used k-means algorithm [36] is that in k-medoids, the cluster prototypes are a subset of the original features, as opposed to averages (which are not actual metabolite features) as in k-means. We chose the number of clusters that allowed us to reach our target $\frac{p}{n}$ ratio, as detailed in the Results section “Modelling to determine the optimal number of k-medoid clusters”. We ran the k-medoids algorithm 10 times, using cosine similarity to define feature similarity from different random initializations and chose the clustering with the best objective function value of the 10 solutions.

### Regression of clusters with bioactivity

The ensemble modeling approach is described in detail elsewhere [26]. To briefly summarize that approach, we perform many random 2/3–1/3 splits of the mass spectrometry fractions into training-test sets. For each split, we generate a predictive model (we originally used both ElasticNets [24] and Random Forests [25], which yielded very similar results) whose parameters are estimated via cross-validation on only the training set. We compute feature importance for this model, but also a model weight proportional to the model’s skill on the test data – which that individual model has never seen. Our ensemble weighted importance values use these model weights to reward models that are good at predicting the test data; the important features from those models that generalize well receive more weight in the final ensemble-average feature importance. There are two substantive changes to this previous ensemble modeling approach that we made for this study. First, the input to the analysis pipeline is not the original set of mass spectrometry features, but the matrix of medoids, formed from the k-medoids clustering. Second, we used L1-penalized regression (the Lasso [38]) for reasons we detail in Results. Other models like Random Forests [25], which we used previously [26], or Support Vector Regression [44] would also be possible; in future work we will explore the effectiveness of these algorithms. K-medoids clustering and ensemble Lasso regression code is publicly available at https://github.com/kevinbrownlab/ceres.

## Results and discussion

### Bioactivity measurement

**Fig 1:**
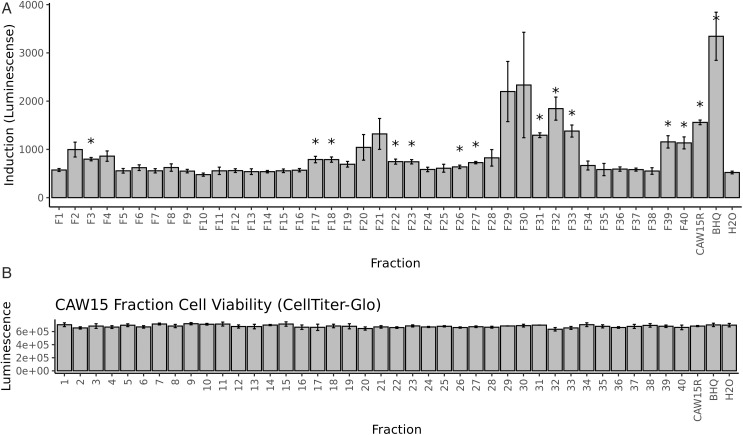
NRF2 Induction and Cell Viability (A) NRF2 activation of each 40 fractions. Each fraction (740 µg/mL) was run in triplicate and statistical significance (* p < 0.05) was tested against the H_2_O control using multiple hypothesis correction (Benjamini Hochberg). (B) Cell viability measured by CellTiter-Glo shows no significant impact to cellular viability after cells were exposed to CAW fractions. BHQ was used as a positive control for Nrf2 activation.

Bioactivity of each fraction was performed using an NRF2/ARE assay. A subset of those fractions showed NRF2 activation compared to the water control (Fig 1A). The starting material CAW-15 (1000 µg/mL, 750 µg/mL, and 500 µg/mL) also showed NRF2 induction and in a dose dependent manner (S2 Fig.). Tert-butyl hydroquinone (BHQ; 43 µg/mL) was used as positive control. Results indicate that the LH-20 fractionation of the water extract of *Centella asiatica* (CAW15) contains multiple chemical components that result in NRF2 activation. Significance was determined by multiple hypothesis corrected t-tests (Benjamini-Hochberg, *p < 0.05) and calculated in R version 4.4.2. No impact on cell viability was observed for any fraction (Fig 1B)**.** The mass and UV absorbance (325 nm) of the original 156 fractions that were combined down to 40 fractions for final analysis are shown in S3 and S4 Figs.

### Mass spectrometry data acquisition

**Fig 2:**
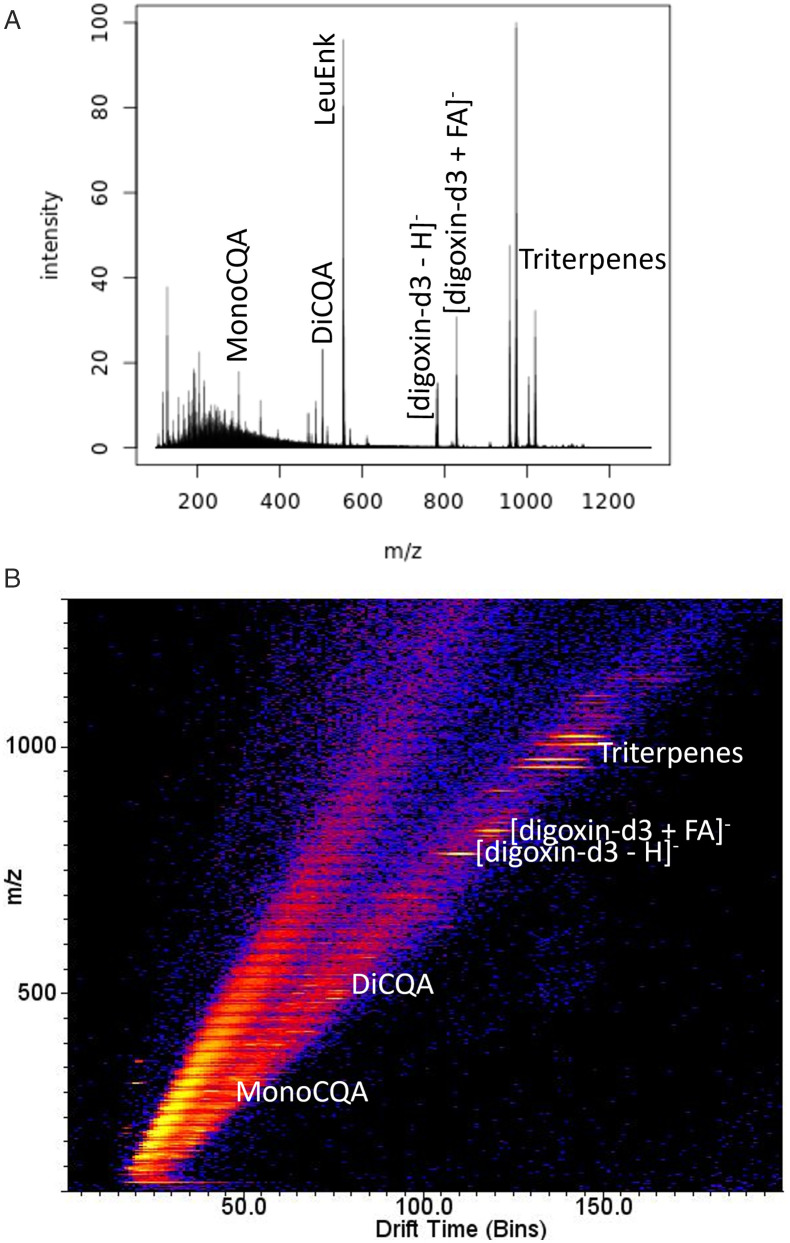
Loop injection ion-mobility mass spectrometry of *Centella asiatica* (A) Mass spectrum from loop injection mass spectrometry analysis of CAW15R, the starting material that was fractionated by LH-20 column chromatography. Leucine enkephalin (LeuEnK) was included as a mass calibrant.(B) Drift time analysis indicated that the ion-mobility increase in peak-capacity enables the deflection or filtration of background noise from the signals of known metabolites in *Centella asiatica.*

Loop injection ion-mobility mass spectrometry analysis of fractions shows a distinct advantage over analyses without ion-mobility. The ion-mobility separation, by increasing peak capacity, reduces the background seen in loop-injection experiments. A traditional mass spectrum view is shown in Fig 2A and much of the background is separated “along the diagonal” of the drift time plot shown in Fig 2B**.** The mass calibrant leucine enkephalin is shown with well-known mass spectrometry signals (verified by authentic standards) from *Centella asiatica* [40,41]. Following lock mass correction and merging of the mass spectrometry data set, each fraction showed a mass error for digoxin-d3 of less than 4 ppm.

Electrospray-ionization mass spectrometry shows adducts, isotopologues, and in-source fragments from each phytochemical. This results in a highly correlated dataset that is usually cleaned up by deisotoping and adduct removing methods. However, those methods often rely on optimum chromatography conditions and coelution of features to detect those redundant features for one chemical. Our loop-injection system, while fast, does not include a separation to leverage for these methods. Ideally, unsupervised clustering techniques should be able to combine such signals, where adducts and isotopes of one chemical species are combined together. By selecting centroid signals, but maintaining knowledge of the cluster membership, we are able to simultaneously reduce the redundant chemical information as well as limiting the number of input variables into the regression model to an optimum.

### K-medoids clustering

K-means [45] and K-medoids [35] are both popular “prototype” clustering algorithms used in unsupervised machine learning to group data into distinct clusters based on some measure of distance. Both basically group points so that points within a group are on average closer to each other than to points in any other group. K-means aims to minimize the variance within clusters by assigning each data point to the nearest centroid, which is the mean of the points in that cluster. The algorithm iteratively updates the centroids and reassigns data points until convergence. K-medoids, on the other hand, uses actual data points as cluster representatives (medoids), rather than the mean. This makes K-medoids more robust to outliers, as it is less sensitive to extreme values. Additionally, it provides the chemist with a chemically measured signal as the representative of a cluster and thus a signal to start with for identification or further experimentation. Finally, k-medoids has Python implementations that run in seconds, which makes it amenable to this high-throughput setting.

### Modelling to determine the optimal number of K-medoid clusters

How many clusters should we use for downstream modeling? Determining the appropriate number of clusters is a ubiquitous problem in cluster analysis, and there are many ways to attempt to determine this number, most of which rely on conceptions of what “good” clusters look like (for a review of cluster quality metrics, see [46]). However, for our purposes, we have a much simpler solution to this problem: *we want to keep as many clusters as possible, while giving the ensemble regression the best chances of success.* Thus, we select the number of clusters to achieve a target $\frac{p}{n}$ ratio. For example, suppose we have 5000 features and 40 measured fractions. If we want a $\frac{p}{n}$ of 1.5, then we want to end up with ~60 medoids. Thus, we would ask for 60 clusters from the original 5000 features.

What should this target $\frac{p}{n}$ be? To estimate this number, we performed simulation studies with synthetic data generated as previously described [39]. We select a desired n (number of samples/fractions) and p (number of predictors/mass spec features). We then generate an n x p data matrix X, which we column standardize. We then choose an integer T between 1 and p. Of the p possible features, only T<p of them will be nonzero, and hence predictive of the simulated bioactivity. Each of the T nonzero betas, chosen at random, are set equal to $\beta_{i}=\epsilon_{i}\left(\text{1}\;+\;\left|a_{i}\right|\right)$, where $\epsilon_{i}$ is a random sign (+1 or –1), and $a_{i}$ is a Gaussian random number with mean zero and variance 1. Finally, we form the assay data as y=Xβ+z , where z is a Gaussian random number with mean zero and variance $\frac{\text{1}}{\text{3}}\sqrt{\frac{T}{n}}$.

We now have a simulated data matrix X and bioactivity y . In the simulated data, while there are p mass spectrometry features (columns of X ) by construction, only a fraction of them are actually predictive of the bioactivity – the rest have no effect in this particular simulated assay. What we would like to know is how effective different regularized regression methods are at recovering the identity (but not necessarily value) of the nonzero βs (bioactive NPs), as a function of $\frac{p}{n}$. This helps us (1) pick the best performing regression method and (2) choose a k for k-Medoids that ensures we still recover a significant fraction of the bioactive NPs.

We proceed by solving our simulated system for the regression coefficients ($\hat{\beta }$) using one of four methods: the ElasticNet [24], the Lasso [38], the Lasso using least angle regression [47], and the Dantzig estimator [39]. Since our primary goal is to obtain nonzero coefficients for *only* the coefficients which were nonzero when we constructed the simulation data, we convert both the vector of known coefficients β and the vector of estimates $\hat{\beta }$ into binary vectors, which have a 1 in any position with a nonzero coefficient and a zero elsewhere. We then compute the adjusted mutual information (AMI) [48] between the two binary vectors. AMI generally ranges from 0 to 1, though it can be negative in small samples. An AMI of zero means that a regression model failed to find *any* of the simulated bioactive NPs (nonzero βs), and an AMI of 1 means the reconstruction was perfect: all βs which were nonzero were also estimated to be nonzero (and no zeros estimated to be nonzero). AMI is particularly suited for our purposes because it is adjusted for chance in imbalanced class labels (many more 0s than 1s).

**Fig 3:**
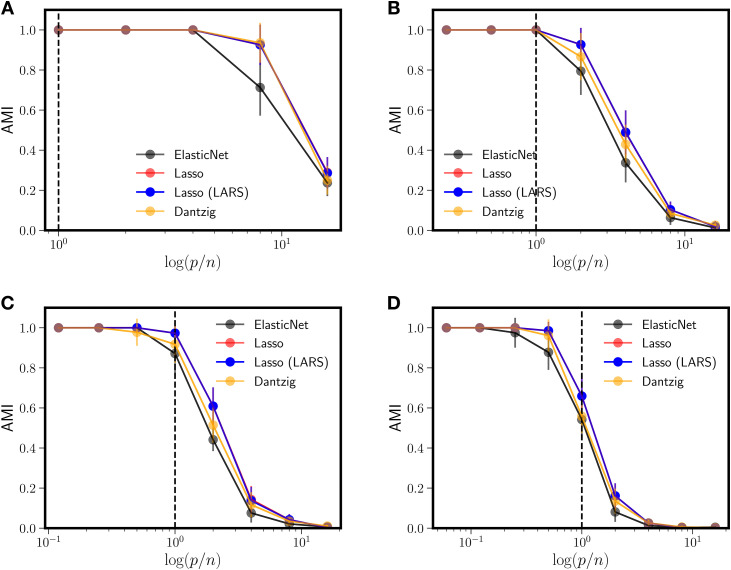
Simulation test for regression models. The four panels show successively increasing ratios of T/p (the ratio of nonzero features to total features): 0.01 (A), 0.05 (B), 0.1 (C), and 0.25 (D). In each case, we show the adjusted mutual information (AMI; see text) at different values of p/n. We mark p/n = 1 with a dotted line; all values greater than this yield a “wide” problem in which we have more variables than samples [49].

Fig 3 shows the results of these simulations. In all cases, we fixed n at 100 and then varied p in multiplicative steps from $\frac{n}{\text{1}\text{6}}$ to 16n . The four different panels show increasing values of $\frac{T}{p}$: 0.01 (panel A), 0.05 (B), 0.1 (C), and 0.25 (D). Recall, $\frac{T}{p}$ represents the fraction of masses that we measure which are associated with the measured bioactivity. Hence, these four cutoffs correspond to 1%, 5%, 10%, and 25% (respectively) of measured peaks being bioactive. We see that solution quality varies strongly with $\frac{T}{p}$. At 1% bioactive masses, we can achieve almost perfect recovery at a $\frac{p}{n}$ as large as 8 and even recover some true peaks at 16. If, on the other hand, 25% of the total masses measured show some correlation with bioactivity, solution quality on any problem size with p>n becomes extremely poor. Of course, we cannot know the true number of bioactive masses. However, we think it reasonable to conclude that in typical natural product bioassays, which focus on specific phenotypic changes and chemical activity, the vast majority of measured compounds in the source material will have no effect. Thus, for this study, we pick $\frac{p}{n}=\text{1}.\text{5}$ as a conservative choice. This ensures good performance even if the true value of $\frac{T}{p}$ is that shown in Fig 3C. This is the number of clusters we will use for working with the NRF2 data subsequently, though in what follows we test the stability of the most bioactive clusters to this choice of cluster number.

### K-medoid clustering and LASSO regression of mass spectrometry and bioassay results

**Fig 4:**
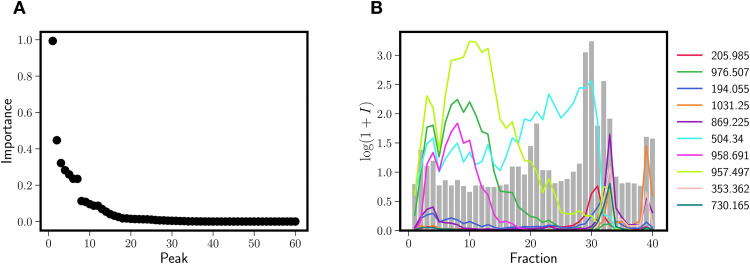
Variable importance and bioactivity in NRF2 induction. A. Variable importance for the 60 clusters (reduced from an original 5,312 peaks). Note the relatively small number of important exemplars. B. Plot of the bioactivity (gray bars) and across-fraction natural log intensity of the top 10 most important exemplars/medoids (colored traces). m/z values for the medoids are denoted in the legend to the right. The gray bars have been rescaled to be visible on the same scale as the across-fraction peak log intensities.

Now we apply our computational pipeline to the NRF2 bioassay data. We begin with 5,312 peaks, a number of features (p) vastly larger than the number of fractions/samples (n). Following the arguments in the previous section, we chose 60 clusters (1.5 x 40) as our reduction target. After k-medoids clustering and ensemble Lasso regression, the variable (cluster) importance is ranked, as described previously [26]. Fig 4A shows sorted importance values for each of the 60 medoids/exemplars (which, when produced by k-medoids, correspond to actual compounds, not cluster averages). Note that, as we have seen repeatedly [26,37], variable importance falls off very quickly making only the first few clusters important in determining the bioactivity. Fig 4B shows the NRF2 activity (gray bars) and the across-fraction natural log peak intensities of the medoids/exemplars for the top ten important clusters. We note that different medoids can be responsible for different phases (early, middle, and late fraction) bioactivity. For example, m/z 1031.250 (orange) is localized mainly to late fractions (with some occurring near fraction 34), while m/z 205.985 (red) is exclusively present in fractions near 30.

Table 1 shows the cluster number, its medoid, and identities for the peaks within that cluster. Identity annotations levels for m/z’s in each cluster, as defined in the Metabolomics Standards Initiative [52], are given in superscript within Table 1. Full cluster m/z’s are shown in S1 Table. Additionally, a supplementary table (S2 Table) including retention time (Ph-3 2 µm, 2.1 x 100 mm) and fragmentation spectra collected on the starting material CAW15 by liquid chromatography quadrupole time-off-flight and data-dependent acquisition is provided. Identities are consistent with previous reports of bioactivity in *Centella asiatica*, verifying the method’s ability to detect bioactive constituents. Caffeoylquinic acid and dicaffeoylquinic acids (clusters 26 and 1) have been previously shown to be protective against amyloid toxicity in a mouse model of Alzheimers disease [50,51]. And, the triterpenes asiatic acid and madacassic acid and their associated glycosides madacassoside and asiaticoside have been linked to a broad range of pharmacological activities [52] as well as the attenuation of skin damage from UV radiation [53].

**Table 1:** Ranked regression results for top clusters including the exemplar m/z and assessment of possible identities in each cluster. Identity annotation levels given in superscript as defined in the Metabolomics Standards Initiative [54]: superscript ^1^ = confirmed identification, supported by retention time and/or MS/MS match of compounds detected in the parent extract compared to authentic reference standard; superscript ^2^ = probable identification based on MS/MS spectral library match of the parent extract without an authentic standard; superscript ^3^ = putative annotation based on accurate mass match to database entries within 4 ppm, without MS/MS or retention time confirmation. MS/MS spectra and retention times for select compounds measured in the parent extract are provided in S2 Table and Supplemental S5 Fig. Full cluster m/z’s and membership are given in S1 Table. Three decimal places are used because initial data is binned to nearest 0.001 prior to clustering and regression analysis.

Cluster	Medoid	Possible Identities of m/z’s in Cluster
**18**	205.985	Possible in-source fragment of an oxidized ellagic acid derivative^3^
**9**	185.024	Quinic Acid^1^, Quercetin 3-O-glucoside^1^, 1-Caffeoyl-5-feruloylquinic acid^3^, Apimaysin^3^, Stachyose^3^
**42**	119.046	Adenine^3^, 3,4-Dihydroxybenzaldehyde^3^, Succinate^3^, Caffeic acid^1^, Ferulic Acid^1^, Muramic acid^3^, 16-hydroxypalmitic acid^3^, Dicaffeoylquinic acids^1^
**26**	515.382	Dicaffeoylquinic acids^1^
**1**	109.025	3-Hydroxycoumarin^3^, Caffeic acid^1^, Quercetin^1^, Aesculin^3^, Caffeoylquinic acids^1^, Asiatic acid^1^, Vincosamide^3^, Madecassic acid^1^, Asiatic acid^3^, Stachyose^3^
**5**	487.332	Asiatic Acid^1^, Madecassic Acid^1^
**38**	327.319	Asiaticoside^1^, Madecassoside^1^
**55**	469.144	Asiaticoside^1^, Madecassoside^1^
**7**	173.202	Caffeoylquinic acids^1^, Aconitic acid^3^, Caffeic acid^1^
**46**	144.042	Quinic Acid^1^, 4-Guanidinobutanoic acid^3^, Ferulic acid^1^, Isovalerylglucoronide^3^, Epigallocatechin^3^, Rutin^2^, Naringin^3^

Interestingly, Cluster 18 contained a single m/z at 205.985 (binned to nearest 0.001 prior to clustering and regression), which is not a previously annotated mass in *Centella asiatica* extracts [37,40,55]. Cluster 18 is shown as a red trace in Fig 4B and is most abundant in fractions 30 and 31. So, we combined those two fractions and analyzed that sample by ultra-performance liquid chromatography tandem mass spectrometry UPLC-MSMS (method described previously in [55]) to collect a retention time and MSMS spectrum of that precursor (S5 Fig.). No identity was found when searching in multiple publically available databases, and *in silico* efforts at annotation gave erroneous results (SIRIUS, [56]); however, it is plausable that it could be a result of the in-source fragmentation of an oxidized ellagic acid derivative, a conjugated quinone that could activate NRF2 (S5 Fig.) [57]. Continued measurement in other *Centella asiatica* studies, fraction enrichment, and/or other NRF2 bioassay experiments will help to elucidate this mass and its associated metadata for future follow-up in subsequent studies [58].

How stable are the predicted clusters? We first address variation for two clusterings with the same $\frac{p}{n}$ (ignoring the subsequent modeling step), and then for two different choices of $\frac{p}{n}$, in which we run the entire pipeline, from clustering to ensemble model. Variation due to random initialization of k-medoids is minimal; in fact, we chose the clustering parameters (described in Methods) to make clustering variability low. If we cluster the NRF2 data twice at $\frac{p}{n}=\text{1}.\text{5}$ and measure the adjusted mutual information [59] between the two clusters, we obtain a value of 0.97 out of a maximum of 1.0, indicating an extremely high degree of overlap between the two clusterings. To test predicted feature stability, we compared the top clusters we obtained from the $\frac{p}{n}=\text{1}.\text{5}$ model (the one we report above) to a second model that uses a more liberal cutoff of $\frac{p}{n}=\text{3}.\text{0}$. S6 Fig shows a heatmap that compares cluster membership between the two sets of the top 10 most important clusters for both models. There is some minor shuffling of importance – hit 5 in the original model has moved to hit 6 in the comparison model, and hit 6 in the original model has moved up to hit 4 in the comparison model. Stability is quite good, especially among the most important features. Hits 1 and 3 are identical in the two models, and hit 2 has now been split into two clusters, one which stays in position 2 in the comparison model, and one which lands at position 10. This kind of splitting is not unexpected, as the comparison model has twice as many clusters (120 compared to 60) as the original model.

**Fig 5:**
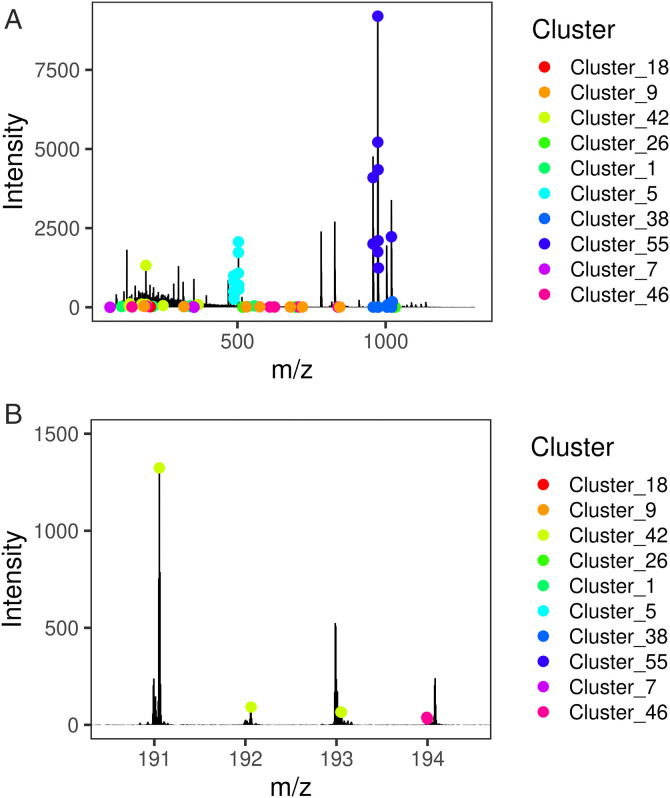
Clusters projected onto the starting material’s (CAW15R) mass spectrum. (A) Signals from the top ten clusters important to bioactivity (as predicted with our regression models) are plotted as a scatter plot on top of a mass spectrum of the starting material used in fractionation. Notable is that the mass spectrometry signals from madecasosside, which shows up as a combination of two spectral features [M-H] ^–^ and [M+COOH]^-^ and the naturally occuring isotopologs are clustered together in Cluster_38, demonstrating that k-medoids effectively clusters isotopologs and adducts of a single metabolite. (B) Shows the detection of low mass and lower signal peaks from Cluster_46 and Cluster_42 from the presense of quinic acid (m/z 191.0561) and ferulic acid (m/z 193.0506).

Importantly, our k-medoids clustering effectively grouped isotopes, adducts, and in-source fragments of a singular metabolite together (Fig 5), as evidenced by the clustering of madecassoside in Cluster_38. This cluster included mass spectrometry signals from both [M-H]^-^ and [M+COOH]^-^ ions (and the associate isotopic abundances), showcasing the algorithm's ability to accurately identify related molecular species. Furthermore, the detection of low-intensity peaks corresponding to quinic acid (m/z 191.0561 reported to alleviate neuroinflamation [60]) and ferulic acid (m/z 193.0506 reported to improve cognitive deficits in Alzheimer’s disease [61]) in Clusters_46 and Cluster_42, respectively, highlighted the success of our approach in identifying key bioactive compounds present in the starting material CAW15R. Chemical redundancy exists in the final 60 clusters used in the regression models, but subsequent interpretation and identification becomes much more straightforward with a limited number of important clusters. These results show that k-medoids is an effective, scalable solution to cluster correlated chemical signals in mass spectrometry data.

This study presents a significant advancement in the field of natural product research by integrating high-throughput *in vitro* assays with cutting-edge mass spectrometry and machine learning techniques. The novel pipeline developed herein, combining k-medoids clustering and ensemble Lasso regression, addressing several longstanding challenges in the fraction-based identification of bioactives in botanical extracts. We reduced the need for analytical chromatographic separation in the early stages of bioactive compound prioritization by utilizing ion-mobility to increase sample mass spectrometry throughout and provide a more untargeted detection platform. CERES is specifically designed to complement conventional bioassay-guided workflows by accelerating the dereplication step; downstream structural validation by LC-MS/MS, isolation, and NMR remains an essential next step for confirmed compound identification. We were able to correlate bioactivity data directly with mass spectrometry signals without specifically relying on pre-existing knowledge of known compounds. This approach allowed us to not only increase the sensitivity for detecting new bioactive molecules but also offers a more comprehensive analysis of the full chemical landscape present in a botanical extract. This novel method has the potential to drastically reduce the time and resources required for detecting specific bioactive compounds while increasing the likelihood of discovering previously overlooked bioactive constituents

The ability to efficiently correlate mass spectrometry data with biological activity provides a powerful tool for drug discovery from complex natural products. One of the key advantages of this approach is its flexibility in dealing with the multifaceted nature of phytochemicals, detecting multiple potentially bioactive components and prioritizing them for subsequent targeted fractionation and purification workflows. Through the clustering of molecular features and subsequent regression modeling, we were able to provide insight into the potential bioactivity of chemical compounds in *Centella asiatica*, both known and novel. This ability to uncover and predict the bioactivity of individual chemical components, even in the face of significant chemical complexity, and with such speed, marks a major step forward in the integration of mass spectrometry and machine learning for natural product analysis. This new approach improves upon previous software performance in that it allows for the detection of lower signal-to-noise signals, provides cluster membership of m/z’s that are useful for further investigation of chemical identity of unknowns and their bioactivity, and the combination of k-medoids and ensemble Lasso improves regression model accuracy and throughput compared to our previous successful approaches [26,37].

As with all metabolomics-based bioactivity correlation approaches, the statistical associations identified by CERES should be interpreted as prioritized candidates for downstream experimental validation, not as confirmed bioactive compounds. Several sources of uncertainty warrant explicit consideration. First, although Lasso regression’s sparsity-inducing penalty reduces false positive associations relative to ordinary least squares, it does not eliminate them – compounds that co-vary with a true bioactive across fractions may receive non-zero regression coefficients. Second, because CERES uses loop-injection without chromatographic separation, all compounds within a fraction co-elute in every injection. Compounds that vary together across fractions are indistinguishable by the regression model alone; follow-up LC-MS/MS analysis (as performed here for m/z 205.985, S5 Fig) is an important next step for resolving this ambiguity in high-priority candidates. Third, the linear additive model assumed by Lasso regression does not account for synergistic interactions between phytochemicals. Observed bioactivity in fractions containing multiple co-eluting compounds may reflect entourage effects [7] rather than the activity of any single molecular species. These considerations define the scope of CERES outputs as a prioritization tool that feeds into, rather than replaces, subsequent experimental validation.

Incorporating ethnobotanical knowledge into modern drug discovery has long been hindered by the time-consuming processes of bioassay-guided fractionation and the difficulty of isolating specific bioactive compounds, particularly those of low abundance. The method described in this study mitigates these challenges, offering a more efficient and scalable approach to identifying and validating bioactive natural products. The elucidation of active components in these traditional medicines is of importance to ensuring their presence in complex botanical preparations, and in the identification of lead compounds for the development of new pharmaceutical drugs. In this latter context of drug discovery, the ability to identify bioactive molecules without the need for their purification allows for more rapid dereplication efforts.

As methodologies such as this become more widely adopted, there could be significant enhancements in the translation of traditional botanical remedies into evidence-based therapeutics. The aim would be to improve our ability to predict and verify bioactivity and/or toxicity of natural products in high throughput, optimizing the safety and bioactivity profiles of botanical supplements for humans. The clustering and ensemble regression approach proposed here could conceivably be applied to larger and more complex bioassays or even *in vivo* animal models which would help address key translational concerns for the integration of botanical products into clinical practice. The potential for this technology to accelerate the development of new, plant-based therapeutics is exciting, marking a promising direction for future research in the field of natural product drug discovery.

## Conclusion

This study introduces an innovative approach (CERES) for the identification of bioactive compounds in complex botanical extracts by combining high-resolution mass spectrometry with advanced machine learning techniques (https://github.com/kevinbrownlab/ceres). By leveraging k-Medoids clustering and ensemble Lasso regression, we have successfully addressed critical challenges in phytochemical analysis, including the complexity of natural product compositions, low-throughput in traditional bioassay-guided fractionation and chromatographic approaches, the “many-to-one” clustering problem of multiple adducts and isotopologues in mass spectrometry data, and the problem of a vast excess of features compared to bioactivity measures causing poor regression model performance. In this proof-of-concept application, this method enhanced the efficiency of discovering bioactive constituents and offers a novel means of correlating chemical features with biological effects. If validated across additional botanical systems and bioassay types, this approach has the potential to accelerate the integration of ethnobotanical knowledge into evidence-based medicine, improving both the safety and efficacy of natural products. It is important to include that this method could be used to detect potentially toxic agents in an extract by using a bioassay with toxicity as its end point. Ultimately, the pipeline developed in this study provides a promising pathway for advancing natural product drug discovery and could significantly contribute to the development of new therapeutics derived from plant-based sources.

Several important limitations of the current study should be noted. The CERES pipeline has been validated on a single botanical system (*Centella asiatica* water extract) using a single bioassay endpoint (NRF2/ARE activation); whether the approach generalizes to botanicals with different chemical complexity profiles or different bioassay types requires independent evaluation. The fractionation and mass spectrometry analysis were performed on a single extract batch (BEN-CAW-15), so inter-batch variability is not characterized here. Compound identities reported in Table 1 are assigned primarily by accurate mass matching to databases; definitive structural confirmation requires isolation and NMR or comprehensive MS/MS characterization. Finally, the linear additive model assumed by ensemble Lasso regression may not fully capture synergistic phytochemical interactions as is currently stands (but could by adding interaction terms to the model). These limitations define the scope of the current work as a proof-of-concept demonstration and identify the validation studies needed before CERES can be routinely deployed in natural product drug discovery programs.

## Supporting information

S1 Fig Workflow schematic of extraction preparation prior to LH-20 fractionation. BEN-CAW-15 is the initial water-extracted material from the plant. CAW15 EI, refers to the water:ethanol insoluble material. CAW15R is the water:ethanol soluble material that then was lyophilized and dissolved in methanol. A small portion of CAW15R is not soluble in methanol and is termed CAW15 W:E MI, being water and ethanol soluble, but not in methanol. The final CAW15RF is the portion of CAW15R that was loaded onto the LH-20 column for fractionation.
(TIFF)

S2 Fig Pre-column fractions of CAW15 (S1 Fig.) show dose dependent activation of NRF2. (TIFF)

S3 Fig Mass of each initial fraction after drying under nitrogen stream. The fractionation resulted in 156 crude fractions. Fractions were dried, weighed, and reconstituted in water and combined to obtain a final 40 fractions of equal concentrations (375 mg/mL).
(TIFF)

S4 Fig UV absorbance (325 nm) of each fraction before drying under nitrogen stream. While the mass of material is focused around the initial peak near fraction 50, later eluting components have significant UV absorbance.
(TIFF)

S5 Fig To obtain enough signal for a MS2 spectrum, fractions 30 and 31 were combined to a concentration 50x more concentrated than the original mass spectrometry fractions run by loop-injection. The sample was analyzed by Ph-3 UPLC separation using a water and methanol gradient as reported previously [S1]. A chromatographic retention time of 5.9 min was recorded as well as the MS2 spectrum of this low abundant chromatographic peak. The negative mass defect of the molecular ion suggests the presence of multiple oxygens, likely for a compound that has NRF2 activity. It is possible that m/z 205.9853 is an in-source fragment of an oxidized ellagic acid derivative and as such, the conjugated quinone could activate NRF2.
(TIFF)

S6 Fig Sensitivity analysis. For each of the top 10 clusters in the p/n = 1.5 model, we computed a normalized intersection with each of the top 10 clusters in the p/n = 3.0 model to compare cluster membership. Specifically, we computed the number of masses in the intersection of the two clusters, divided by the size of the smaller cluster. If this number is 1.0, it means every mass in the smaller cluster is contained in the larger clusters – along with potentially other masses as well. While there is some shuffling of importance and splitting of larger clusters in the p/n = 1.5 model into smaller ones in the p/n = 3.0 model, we note the strong agreement among the most important peaks. For example the top hit (a singleton cluster) is exactly the same compound in both models.
(TIFF)

S1 Table Cluster m/z membership for k-Medoids clusters most associated with bioactivity as determined by Lasso regression. Included are possible identities of chemical components previously found in *Centella asiatica [S1, S2].*
(DOCX)

S2 Table Top hits masses detected with liquid chromatography mass spectrometry acquisition and associated mass spectrometry fragmentation data. (CSV)

S1 File References for supporting information (S1 and S2). (DOCX)
