# Supplementary material for: CERES: Cluster‑enabled regression of extract signatures for discovery of NRF2 activators in Centella asiatica by ion-mobility mass spectrometry, k-medoids clustering and ensemble Lasso regression
Source: PLoS One. 2026 Aug 6;21(8):e0354664. doi: 10.1371/journal.pone.0354664 (PMC13446660; doi:10.1371/journal.pone.0354664)
Supplement: Supplementary file 1 [file pone.0354664.s007.docx]

Cluster 18 m/z's	Possible Identities
205.985	Possible in-source fragment of an oxidized ellagic acid derivative

Cluster 9 m/z's	Possible Identities
185.024	N/A
185.03	N/A
191.233	Quinic Acid
239.194	N/A
239.289	N/A
319.063	N/A
498.1	N/A
498.11	N/A
498.119	N/A
499.098	Quercetin 3-O-glucoside [M+Cl]$^-$
499.108	Quercetin 3-O-glucoside [M+Cl]$^-$
499.118	Quercetin 3-O-glucoside [M+Cl]$^-$
500.107	Quercetin 3-O-glucoside [M+1+Cl]$^-$
500.117	Quercetin 3-O-glucoside [M+1+Cl]$^-$
530.13	N/A
530.141	N/A
535.116	N/A
563.128	N/A
563.138	N/A
571.132	N/A
571.143	N/A
575.129	1-Caffeoyl-5-feruloylquinic acid [M+FA]$^-$
575.14	1-Caffeoyl-5-feruloylquinic acid [M+FA]$^-$
576.128	1-Caffeoyl-5-feruloylquinic acid [M+1+FA]$^-$
576.139	1-Caffeoyl-5-feruloylquinic acid [M+1+FA]$^-$
576.149	1-Caffeoyl-5-feruloylquinic acid [M+1+FA]$^-$
605.104	Apimaysin [M+FA]-
605.115	Apimaysin [M+FA]-
607.132	N/A
629.14	N/A
629.151	N/A
641.145	N/A
641.156	N/A
659.156	N/A
678.17	N/A
678.182	N/A
679.164	N/A
679.175	N/A
683.155	N/A
683.167	N/A
684.175	N/A
701.152	Stachyose [M+Cl]$^-$
701.187	Stachyose [M+Cl]$^-$
702.162	Stachyose [M+1+Cl]$^-$
702.173	Stachyose [M+1+Cl]$^-$
702.185	Stachyose [M+1+Cl]$^-$
715.485	N/A
715.497	N/A
718.172	N/A
718.184	N/A
719.159	N/A
719.171	N/A
720.17	N/A
720.182	N/A
720.193	N/A
763.165	N/A
781.199	N/A
845.22	N/A
863.215	N/A
863.228	N/A
883.222	N/A
895.205	N/A
895.218	N/A
895.231	N/A
937.225	N/A

Cluster 42 m/z's	Possible Identities
119.046	N/A
119.051	N/A
134.03	Adenine
134.035	Adenine
134.04	Adenine
137.019	3,4-Dihydroxybenzaldehyde
163.038	Succinate [M+FA]$^-$
175.035	N/A
175.041	N/A
176.005	N/A
176.011	N/A
177.018	N/A
177.024	N/A
178.022	Caffeic acid [M-2H]$^-$
192.064	Ferulic acid [M-2H]$^-$
193.043	Ferulic acid [M-H]$^-$
193.049	Ferulic acid [M-H]$^-$
193.055	Ferulic acid [M-H]$^-$
193.062	Ferulic acid [M-H]$^-$
193.068	Ferulic acid [M-H]$^-$
194.049	Ferulic acid [M+1-H]$^-$
194.055	Ferulic acid [M+1-H]$^-$
194.061	Ferulic acid [M+1-H]$^-$
205.049	6-Amino-9H-purine-9-propanoic acid [M-2H]$^-$
221.043	N/A
247.059	N/A
249.074	Muramic acid [M-2H]-
249.081	Muramic acid [M-2H]-
249.088	Muramic acid [M-2H]-
250.078	Muramic acid [M-H]-
283.105	N/A
307.076	16-hydroxypalmitic acid [M+Cl]$^-$
337.087	3,5-Dihydroxy-2-methylphenyl-beta-D-glucopyranoside [M+Cl]-
367.093	N/A
367.102	N/A
367.11	N/A
368.093	N/A
368.102	N/A
368.11	N/A
369.094	N/A
369.103	N/A
371.084	N/A
371.092	N/A
372.089	N/A
372.098	N/A
403.107	N/A
403.116	N/A
561.154	Dicaffeoylquinic Acids [M+FA]$^-$
561.164	Dicaffeoylquinic Acids [M+FA]$^-$
707.199	N/A

Cluster 26 m/z's	Possible Identities
515.382	Dicaffeoylquinic acids [M-H]$^-$
515.402	Dicaffeoylquinic acids [M-H]$^-$
515.452	Dicaffeoylquinic acids [M-H]$^-$
515.512	Dicaffeoylquinic acids [M-H]$^-$
518.122	N/A
518.132	N/A
695.142	N/A
695.154	N/A
695.165	N/A
695.177	N/A
696.159	N/A
696.171	N/A
1031.236	N/A
1031.25	N/A
1031.264	N/A
1032.249	N/A
1032.263	N/A

Cluster 1 m/z's	Possible Identities
109.025	N/A
109.029	N/A
132.018	N/A
136.041	N/A
161.091	3-Hydroxycoumarin [M-H]$^-$
162.022	3-Hydroxycoumarin [M+1-H]-
215.051	Caffeic acid [M-H]$^-$
337.079	Quercetin [M+Cl]$^-$
339.045	Aesculin [M-H]$^-$
339.053	Aesculin [M-H]$^-$
339.061	Aesculin [M-H]$^-$
341.073	N/A
353.263	Caffeoylquinic acids [M-H]$^-$
355.078	Caffeoylquinic acids [M-H]$^-$
356.094	Caffeoylquinic acids [M+1-H]$^-$
357.071	Caffeoylquinic acids [M+2-H]$^-$
359.086	N/A
439.078	3,5-Dihydroxyphenyl 1-O-(6-O-galloyl-beta-D-glucopyranoside) [M-H]$^-$
487.119	Asiatic acid [M-H]$^-$
487.129	Asiatic acid [M-H]$^-$
489.114	N/A
497.113	Vincosamide [M-H]$^-$
503.11	Madecassic acid [M-H]$^-$
513.097	N/A
513.107	N/A
513.117	N/A
517.145	N/A
519.11	N/A
531.11	N/A
531.12	N/A
533.111	Asiatic acid [M+FA]$^-$
533.141	Asiatic acid [M+FA]$^-$
557.144	N/A
558.137	N/A
559.131	N/A
641.134	N/A
695.189	N/A
701.14	Stachyose [M+Cl]$^-$
705.161	N/A
709.181	N/A
717.151	N/A
867.185	N/A
867.198	N/A
867.211	N/A
867.224	N/A
868.205	N/A
869.2	N/A
869.213	N/A
869.225	N/A
869.238	N/A
870.208	N/A
870.221	N/A
870.233	N/A
909.21	N/A
909.223	N/A
911.22	N/A
911.233	N/A

Cluster 5 m/z's	Possible Identities
487.332	Asiatic acid [M-H]$^-$
487.342	Asiatic acid [M-H]$^-$
487.351	Asiatic acid [M-H]$^-$
488.339	Asiatic acid [M+1-H]$^-$
488.348	Asiatic acid [M+1-H]$^-$
503.326	Madecassic Acid [M-H]$^-$
503.336	Madecassic Acid [M-H]$^-$
503.346	Madecassic Acid [M-H]$^-$
503.464	Madecassic Acid [M-H]$^-$
503.612	Madecassic Acid [M-H]$^-$
504.34	Madecassic Acid [M+1-H]$^-$
504.349	Madecassic Acid [M+1-H]$^-$

Cluster 38 m/z's	Possible Identities
327.319	N/A
469.419	N/A
957.687	Asiaticoside [M-H]$^-$
957.891	Asiaticoside [M-H]$^-$
957.958	Asiaticoside [M-H]$^-$
957.972	Asiaticoside [M-H]$^-$
958.053	Asiaticoside [M+1-H]$^-$
958.067	Asiaticoside [M+1-H]$^-$
958.243	Asiaticoside [M+1-H]$^-$
958.257	Asiaticoside [M+1-H]$^-$
958.379	Asiaticoside [M+1-H]$^-$
958.677	Asiaticoside [M+1-H]$^-$
958.691	Asiaticoside [M+1-H]$^-$
958.894	Asiaticoside [M+1-H]$^-$
959.695	Asiaticoside [M+2-H]$^-$
973.893	Madecassoside [M-H]$^-$
973.962	Madecassoside [M-H]$^-$
974.057	Madecassoside [M+1-H]$^-$
974.071	Madecassoside [M+1-H]$^-$
974.249	Madecassoside [M+1-H]$^-$
974.263	Madecassoside [M+1-H]$^-$
974.386	Madecassoside [M+1-H]$^-$
974.399	Madecassoside [M+1-H]$^-$
974.687	Madecassoside [M+1-H]$^-$
974.892	Madecassoside [M+1-H]$^-$
975.686	Madecassoside [M+2-H]$^-$
975.699	Madecassoside [M+2-H]$^-$
975.891	Madecassoside [M+2-H]$^-$
1003.699	Asiaticoside [M-FA]$^-$
1003.893	Asiaticoside [M-FA]$^-$
1003.907	Asiaticoside [M-FA]$^-$
1004.699	Asiaticoside [M+1-FA]$^-$
1006.519	N/A
1019.7	Madecassoside [M-H]$^-$
1019.896	Madecassoside [M-H]$^-$
1019.91	Madecassoside [M-H]$^-$
1019.98	Madecassoside [M-H]$^-$
1020.078	Madecassoside [M+1-H]$^-$
1020.092	Madecassoside [M+1-H]$^-$
1020.694	Madecassoside [M+1-H]$^-$
1020.904	Madecassoside [M+1-H]$^-$
1021.703	Madecassoside [M+2-H]$^-$
1022.529	Madecassoside [M+3-H]$^-$

Cluster 55 m/z's	Possible Identities
469.144	N/A
827.44	N/A
827.453	N/A
957.484	Asiaticoside [M-H]$^-$
957.497	Asiaticoside [M-H]$^-$
957.511	Asiaticoside [M-H]$^-$
958.487	Asiaticoside [M+1-H]$^-$
958.501	Asiaticoside [M+1-H]$^-$
973.483	Madecassoside [M-H]$^-$
973.497	Madecassoside [M-H]$^-$
973.51	Madecassoside [M-H]$^-$
974.482	Madecassoside [M+1-H]$^-$
974.495	Madecassoside [M+1-H]$^-$
974.509	Madecassoside [M+1-H]$^-$
975.494	Madecassoside [M+2-H]$^-$
1019.491	Madecassoside [M-H]$^-$
1019.504	Madecassoside [M-H]$^-$
1020.498	Madecassoside [M+1-H]$^-$

Cluster 7 m/z's	Possible Identities
173.202	Aconitic acid [M-H]$^-$
179.194	Caffeic acid [M-H]$^-$
353.304	Caffeoylquinic acids [M-H]$^-$
353.321	Caffeoylquinic acids [M-H]$^-$
353.362	Caffeoylquinic acids [M-H]$^-$
353.412	Caffeoylquinic acids [M-H]$^-$
353.42	Caffeoylquinic acids [M-H]$^-$
353.535	Caffeoylquinic acids [M-H]$^-$
353.609	Caffeoylquinic acids [M-H]$^-$

Cluster 46 m/z's	Possible Identities
144.042	4-Guanidinobutanoic acid {M-H]$^-$
191.306	Quinic Acid {M-H]$^-$
191.342	Quinic Acid {M-H]$^-$
191.348	Quinic Acid {M-H]$^-$
191.373	Quinic Acid {M-H]$^-$
191.451	Quinic Acid {M-H]$^-$
192.174	Quinic Acid {M+1-H]$^-$
192.180	Quinic Acid {M+1-H]$^-$
192.186	Quinic Acid {M+1-H]$^-$
192.265	Quinic Acid {M+1-H]$^-$
192.313	Quinic Acid {M+1-H]$^-$
192.320	Quinic Acid {M+1-H]$^-$
192.332	Quinic Acid {M+1-H]$^-$
193.141	Ferulic Acid {M-H]$^-$
193.183	Ferulic Acid {M-H]$^-$
193.189	Ferulic Acid {M-H]$^-$
193.22	Ferulic Acid {M-H]$^-$
193.226	Ferulic Acid {M-H]$^-$
193.263	Ferulic Acid {M-H]$^-$
193.946	N/A
193.952	N/A
193.994	N/A
194.000	N/A
276.063	Isovalerylglucuronide [M-2H]$^-$
351.068	Epigallocatechin [M+FA]$^-$
553.117	N/A
553.127	N/A
553.137	N/A
553.148	N/A
593.134	N/A
593.145	N/A
609.132	Rutin [M-H]$^-$
609.143	Rutin [M-H]$^-$
609.153	Rutin [M-H]$^-$
609.164	Rutin [M-H]$^-$
610.149	Rutin [M+1-H]$^-$
610.16	Rutin [M+1-H]$^-$
625.123	Naringin [M+FA]$^-$
625.134	Naringin [M+FA]$^-$
625.145	Naringin [M+FA]$^-$
625.156	Naringin [M+FA]$^-$
626.132	Naringin [M+1+FA]$^-$
626.143	Naringin [M+1+FA]$^-$
626.154	Naringin [M+1+FA]$^-$

Cluster 46 m/z's continued	Possible Identities
707.175	N/A
707.187	N/A
708.178	N/A
708.19	N/A
714.15	N/A
714.161	N/A
714.173	N/A
714.185	N/A
714.196	N/A
715.145	N/A
715.157	N/A
715.169	N/A
715.181	N/A
715.192	N/A
716.154	N/A
716.165	N/A
716.177	N/A
716.189	N/A
717.163	N/A
717.174	N/A
717.186	N/A
728.177	N/A
729.159	N/A
729.171	N/A
729.183	N/A
729.195	N/A
729.206	N/A
730.165	N/A
730.177	N/A
730.189	N/A
730.201	N/A
757.172	N/A
757.184	N/A
757.196	N/A
839.205	N/A
839.217	N/A
