# Supplementary material for: CERES: Cluster‑enabled regression of extract signatures for discovery of NRF2 activators in Centella asiatica by ion-mobility mass spectrometry, k-medoids clustering and ensemble Lasso regression
Source: PLoS One. 2026 Aug 6;21(8):e0354664. doi: 10.1371/journal.pone.0354664 (PMC13446660; doi:10.1371/journal.pone.0354664)
Supplement: Supplementary file 2 [file pone.0354664.s009.docx]

**References for supporting information (S1 and S2):**

S1.	Alam MN, Marney L, Yang L, Choi J, Cerruti N, Techen N, et al. Clustering of chemical profiles of Centella asiatica cultivars, grown in greenhouses, allows grouping of metabolites with similar production trends. Industrial Crops and Products. 2025;231:121159. doi: <https://doi.org/10.1016/j.indcrop.2025.121159>.

S2.	Alcazar Magana A, Vaswani A, Brown KS, Jiang Y, Alam MN, Caruso M, et al. Integrating High-Resolution Mass Spectral Data, Bioassays and Computational Models to Annotate Bioactives in Botanical Extracts: Case Study Analysis of C. asiatica Extract Associates Dicaffeoylquinic Acids with Protection against Amyloid-beta Toxicity. Molecules. 2024;29(4). Epub 20240213. doi: 10.3390/molecules29040838. PubMed PMID: 38398590; PubMed Central PMCID: PMCPMC10892090.
